# Recurrent myocarditis in a young female with a desmoplakin gene variant: a case report and literature review

**DOI:** 10.3389/fcvm.2026.1750721

**Published:** 2026-04-23

**Authors:** Chenxi Liu, Wen Wen, Mingyue Qu, Zhitao Jin, Jiaojiao Xu, Xiaojiong Lu, Zheng Zhang

**Affiliations:** 1 Chinese People’s Liberation Army Rocket Force Characteristic Medical Center, Beijing, China; 2 The Graduate Training Base of Jinzhou Medical University, Chinese People’s Liberation Army Rocket Force Characteristic Medical Center, Beijing, China

**Keywords:** desmoplakin gene, gene variant, recurrent myocarditis, troponin, WES

## Abstract

**Background:**

Recurrent myocarditis is commonly associated with infectious or immune etiologies. This case suggests a potential association between a *DSP* gene variant and recurrent myocarditis, highlighting a new direction for etiological investigation in such patients.

**Case presentation:**

We present a case of a young female admitted with chest pain and recurrent elevation of serum troponin. Cardiac magnetic resonance imaging (cMRI) revealed late gadolinium enhancement (LGE), indicating myocardial injury. No viral or other infectious causes were identified. Next-generation sequencing detected a heterozygous *DSP* variant (c.2297G > C, p.Ser766Thr), which was confirmed by Sanger sequencing. This finding suggests that the recurrent myocarditis may be associated with the identified *DSP* gene variant.

**Conclusion:**

This case indicates that genetic screening should be considered in patients with recurrent myocarditis. The *DSP* gene should be included in relevant genetic screening panels to facilitate precise diagnosis and inform long-term management strategies.

## 1 Introduction

The typical clinical presentation of myocarditis in young patients includes chest pain and elevated troponin levels. The condition is most frequently attributed to viral infections or autoimmune dysregulation (1, 2). However, the recurrence of myocarditis without a clear infectious cause has shifted focus toward underlying genetic susceptibilities as a potential explanation. The *desmoplakin (DSP)* gene encodes desmoplakin, a core component of desmosomes in the cardiac intercalated disc, which plays a critical role in maintaining mechanical integrity (3). Previous studies have established that individuals with pathogenic *DSP* variants typically present with a clinical phenotype dominated by arrhythmias and dilated cardiomyopathy (4, 5). We report a case of recurrent myocarditis in the absence of conventional triggers. A comprehensive diagnostic workup, including virological and autoimmune serology, returned negative. Given the idiopathic and recurrent nature, genetic testing was pursued. Whole-exome sequencing (WES) identified a heterozygous missense variant in the *DSP* gene (c.2297G > C, p. Ser766Thr), which was subsequently validated by Sanger sequencing. We report this case to supplement the emerging literature on *DSP*-associated inflammatory phenotypes, hypothesizing that the mutation may be associated with recurrent myocarditis.

## 2 Case presentation

### 2.1 Clinical presentation and diagnosis

**Figure 1:**
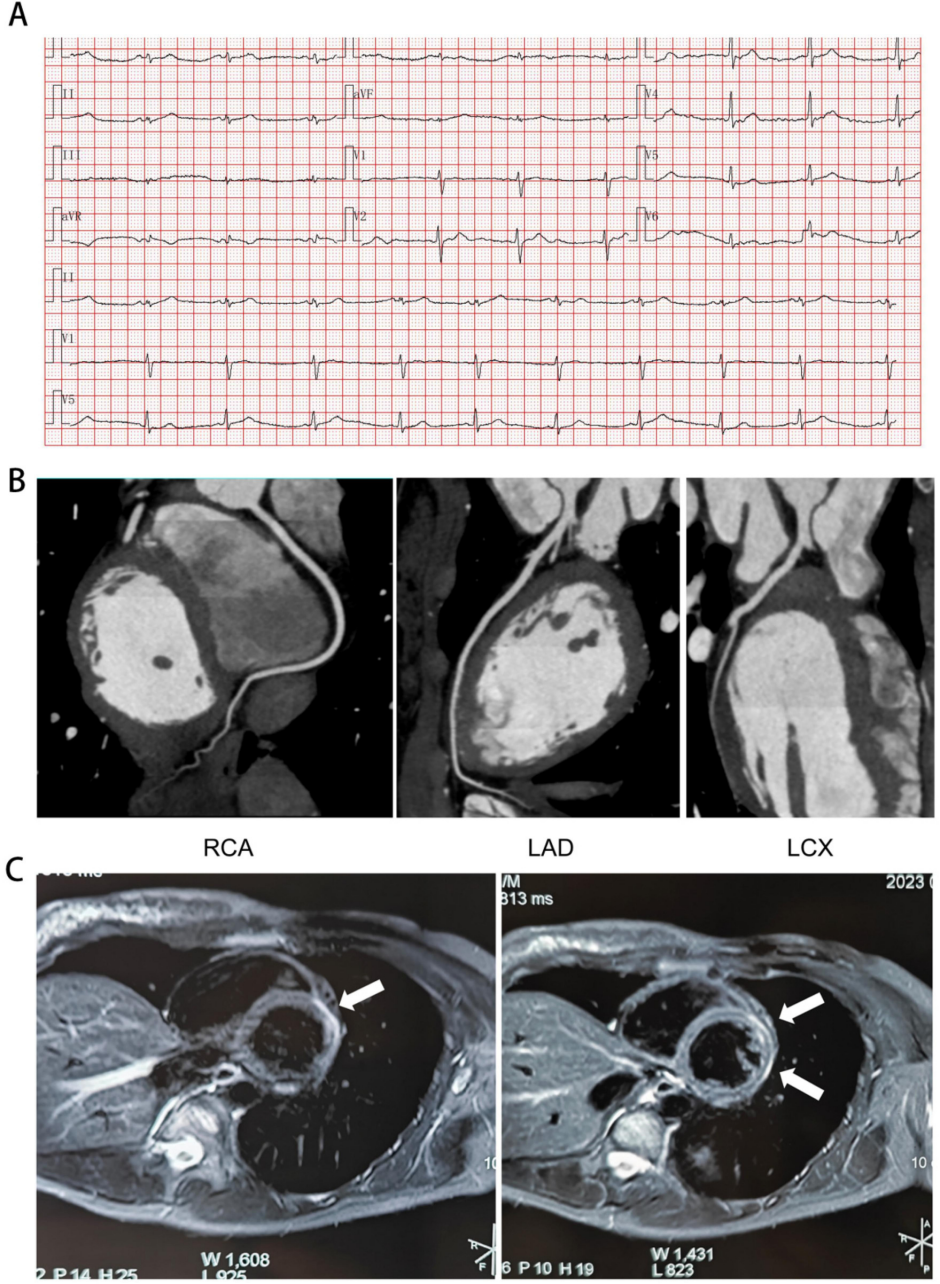
Electrocardiogram and imaging results. **(A)** Electrocardiogram demonstrated sinus rhythm and low QRS voltage in the limb leads. **(B)** Absence of plaque in the RCA, LAD and LCX on CCTA. **(C)** Late gadolinium enhancement in the left ventricular anterior and anterolateral walls on a cardiac magnetic resonance short-axis view (white arrow). RCA, right coronary artery; LAD, left anterior descending artery; LCX, left circumflex artery.

A 23-year-old female patient was presented to the hospital because of chest pain. 12-lead resting Electrocardiogram (ECG) demonstrated sinus rhythm at 60 bpm. Notable findings included low voltage QRS complexes (≤5 mm) in all limb leads. The precordial leads were unremarkable, with normal QRS morphology and duration, and no ST-T abnormalities or QT prolongation. Coronary computed tomography angiography (CCTA) demonstrated no evidence of atherosclerotic plaque in the left circumflex artery (LCX), right coronary artery (RCA) and left anterior descending artery (LAD), which excluded acute coronary syndrome (ACS). Furthermore, computed tomography angiography (CTA) of the thoracic aorta and abdomen showed no abnormalities, excluding an acute aortic syndrome (AAS). Laboratory investigations revealed significantly elevated troponin levels in the absence of clinical evidence of infection. Specifically, white blood cell count (WBC) was 7.69 × 10⁹/L, neutrophil percentage 52.7%, high-sensitivity C-reactive protein (hs-CRP) 0.22 mg/L, procalcitonin 0.039 ng/mL, and interleukin-6 (IL-6) 4.07 pg/mL, all within normal ranges. Cardiac magnetic resonance imaging (cMRI) demonstrated no abnormalities at this initial evaluation. Three months after the initial presentation, the patient was admitted to the emergency department because of severe recurrent chest pain following strenuous physical activity. The initial evaluation in the emergency department revealed a significant elevation in high-sensitivity cardiac troponin (hs-cTn), which rose markedly during hospitalization, peaking at 9,333.8 ng/L. ECG revealed normal sinus rhythm. cMRI demonstrated late gadolinium enhancement (LGE) in the anterior and anterolateral walls of the left ventricle (Figure 1). This subepicardial LGE pattern fulfilled the revised Lake Louise Criteria for CMR-based diagnosis of myocarditis. Eight months later, the patient was admitted to the emergency department because of persistent chest pain following an upper respiratory tract infection. Laboratory testing showed a significant rise in cardiac troponin, with subsequent cMRI confirming myocardial injury through the identification of subepicardial LGE. Given the recurrent episodes of myocarditis marked by troponin elevation, genetic testing was initiated to investigate a potential heritable cause.

**Figure 2:**
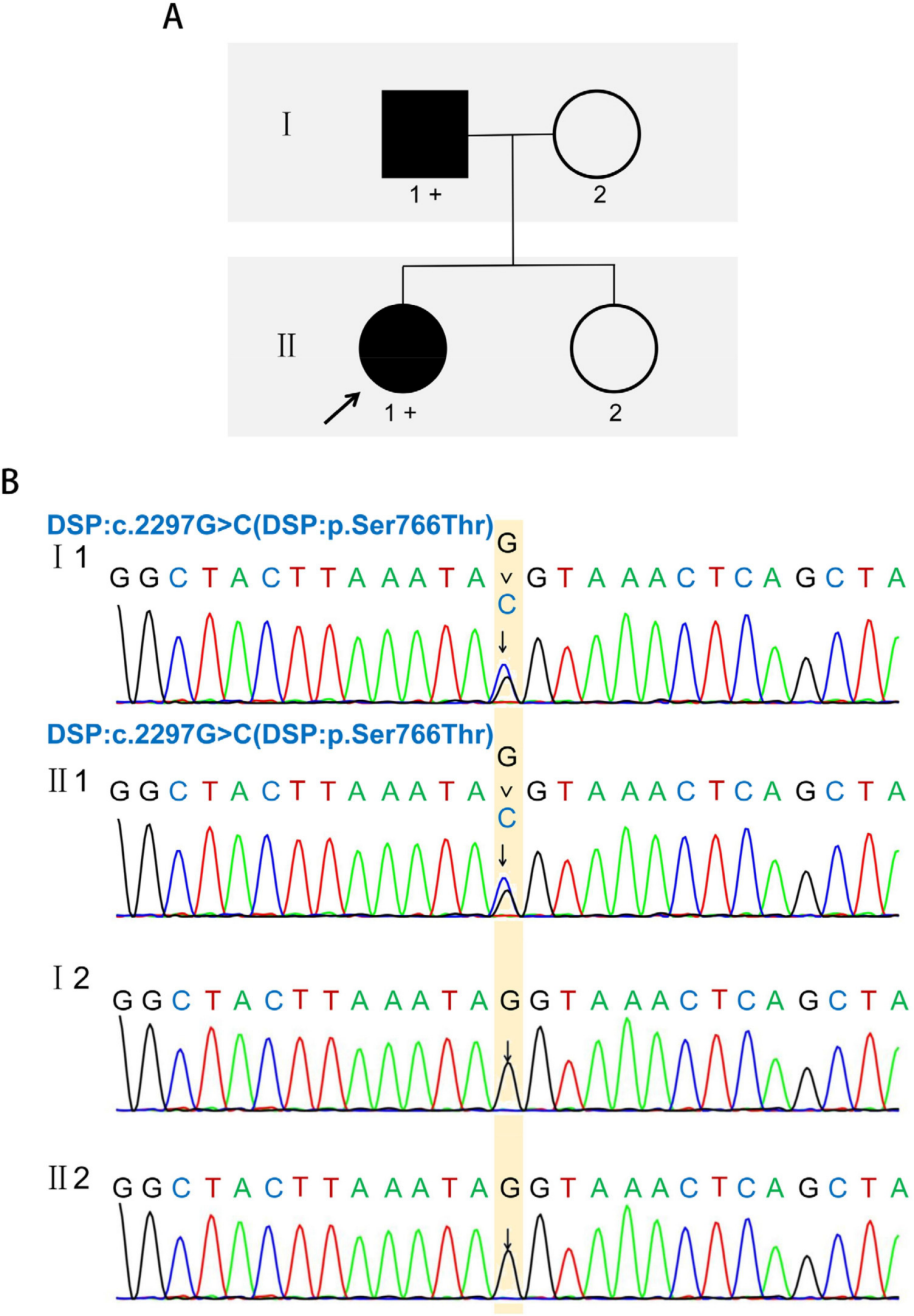
Pedigree chart of the proband and Sanger sequencing results. **(A)** Pedigree chart. **(B)** Sanger sequencing results.

Genetic analysis by WES of peripheral blood, focused on the genes associated with inherited cardiomyopathies and arrhythmias. This analysis identified a heterozygous missense variant in the *DSP* gene: c.2297G > C, resulting in a substitution of serine by threonine at codon 766 (p.Ser766Thr). This was the only variant of interest found in the analyzed gene set. This variant was confirmed by Sanger sequencing, which validated the presence of the c.2297G > C alteration in the proband (Figure 2). Subsequent family segregation analysis revealed that the identical heterozygous *DSP* missense variant was identified in the proband's father (Ⅰ1), whereas it was absent in both the mother (Ⅰ2) and the sister (Ⅱ2), who presented with no symptoms of chest pain or elevated cardiac troponin levels. However, the father (Ⅰ1) declined to undergo cMRI; therefore, imaging data were not available for this individual. According to the American College of Medical Genetics and Genomics (ACMG) guidelines, the variant was classified as a Variant of Uncertain Significance (VUS) based on criteria PM2 (absent from major population databases) and PP3 (multiple *in silico* predictions support a deleterious effect) (6).

**Figure 3:**
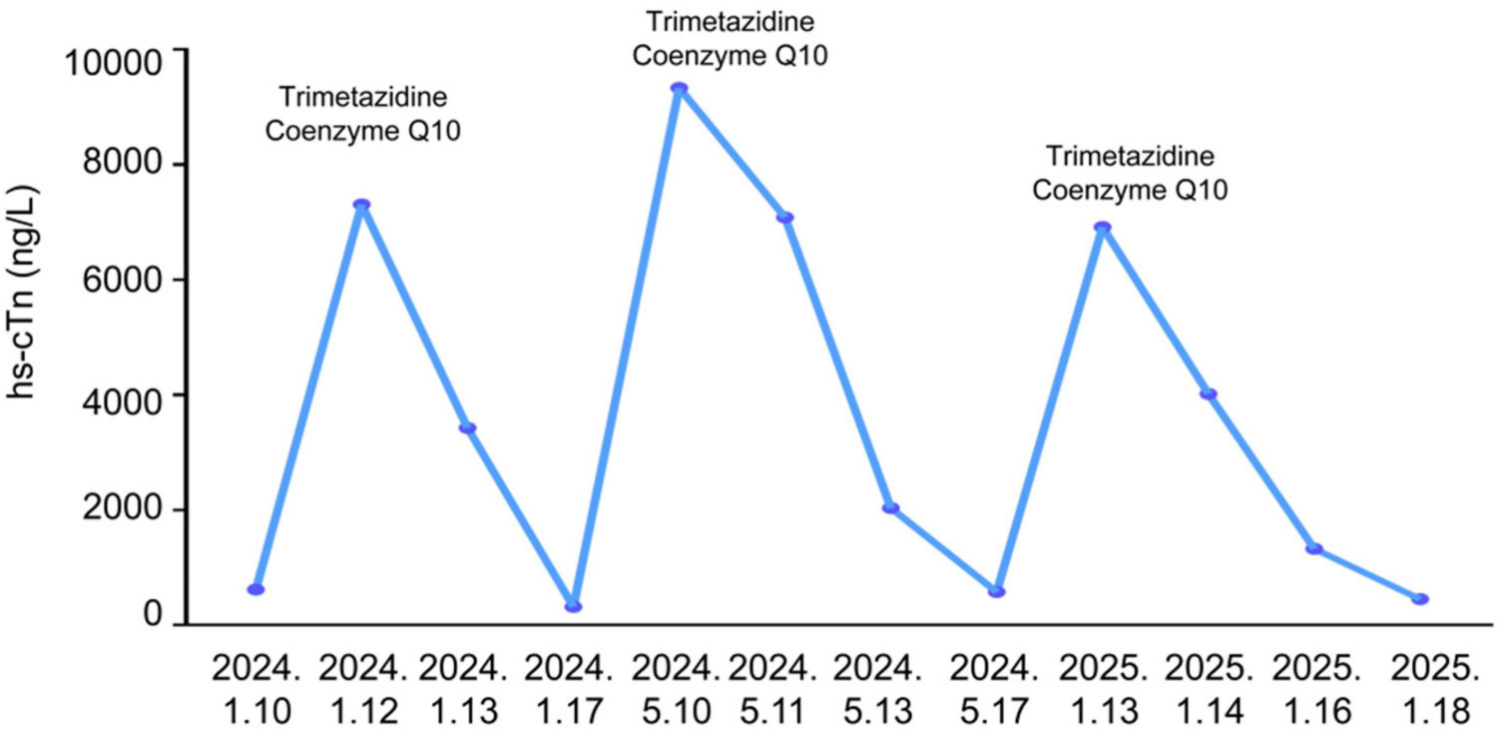
Timeline of elevated high-sensitivity troponin in the patient.

The patient was managed supportively. Trimetazidine and coenzyme Q10 were given as adjunctive metabolic therapy in consideration of possible inflammation-induced myocardial energy dysfunction. Subsequently, troponin levels normalized (Figure 3).

### 2.2 Follow up

During hospitalization, the patient was treated with combination therapy trimetazidine and coenzyme Q10, which was followed by disappearance of chest pain and normalization of serum troponin levels. However, it must be acknowledged that this clinical improvement may reflect the natural course of the disease rather than a direct therapeutic effect of the intervention. At the 11-month follow-up, echocardiography confirmed preserved cardiac function, troponin levels remained within the normal range, and no major adverse cardiac events (MACE) were observed. 24-hour Holter monitoring performed during admission and follow-up revealed sinus rhythm with a total of 98,924 beats and 347 premature ventricular complexes. No non-sustained or sustained ventricular tachycardia was detected, and no significant bradyarrhythmias or conduction abnormalities were observed. The patient is scheduled to undergo annual clinical follow-up, including ECG, echocardiography, and Holter monitoring. In the event of symptom recurrence, CMR imaging will be performed.

## 3 Discussion

We report a case of a 23-year-old female patient who presented with recurrent chest pain and elevated troponin levels. Serologic evaluation for viral and autoimmune etiologies was negative. CCTA demonstrated no coronary plaque, effectively excluding ACS; concurrently, thoracic and abdominal CTA was unremarkable, ruling out AAS. cMRI demonstrated characteristic findings of myocardial inflammation, notably the presence of LGE within the subepicardial region of the left ventricular anterior and anterolateral wall, consistent with a diagnosis of CMR-based myocarditis per revised Lake Louise criteria. WES was performed for genetic screening and identified a heterozygous c.2297G > C (p. Ser766Thr) variant in the *DSP* gene, and the variant was confirmed by Sanger sequencing. The variant is absent from major population databases (1000 Genomes Project, ESP6500, and ExAC), supporting its rarity. Based on the ACMG guidelines, this variant was classified as uncertain significance due to the PM2 Supporting and PP3.

Prior cases of myocarditis associated with mutations in the *DSP* gene have been reported in the literature. It is critical to note that these reported cases predominantly involve protein-truncating variants (nonsense, frameshift, splice-altering), which are definitively pathogenic (Table 1). These six reported cases showed a broad age range (17–49 years) and variable comorbidities; some had diabetes, obesity, or a family history of myocarditis, while others had none. Most patients (5/6) presented with acute chest pain, accompanied by other symptoms such as dyspnea, palpitations, syncope, or ventricular tachycardia. Left ventricular impairment varied widely, with two patients exhibiting severe systolic dysfunction (LVEF as low as 15%). Elevated troponin was observed in four patients, and two experienced recurrent unexplained troponin elevations during follow-up. All previously reported cases carried pathogenic *DSP* mutations and exhibited myocarditis-related phenotypes on cMRI, characterized by subepicardial LGE. In the present case, cMRI revealed LGE involving the anterior and anterolateral walls. The patient experienced 3 distinct episodes of troponin elevation within an 11-month period, and the observed phenotype was similar to that documented in 4 previously reported cases. Notably, the variant identified in this patient was a heterozygous missense mutation, which is fundamentally distinct from the previously documented pathogenic truncating *DSP* mutations. Therefore, this case suggests that some *DSP* missense variants may also be associated with myocarditis; however, causality remains unconfirmed.

**Table 1:** Literature review.

Literature	Case	Sex	Age at Onset	Variant type and position	Family history	Trigger	Symptoms					cMRI	Echocardiogram			
							Chest pain	Palpitation	VT	Shortness of breath	Syncope		Cardiac structural abnormalities	LVDd (mm)	LVEF (%)	Pericardial effusion
Kissopoulou et al. (15)	Elder twin brother (Proband)	Male	17	Nonsense; c.2521_2522del p. Gln841Aspfs*9	−	1. Influenza	+	−	−	−	−	Subepicardial LGE in the LV	Normal	−	46	−
						2. Vigorous activity										
	Younger twin brother	Male	18		−	1. Tonsillitis	+	−	−	−	−	Subepicardial LGE in the LV	Normal	−	52	−
						2. Vigorous activity										
Rezaei Bookaniet al. (16)	Case 1	Female	21	c.1267-2A > G	Arrhythmogenic myocarditis in patient's brother	COVID 19	+	−	+	−	−	LGE in inferior, inferolateral, anterolateral, and anterior walls in the LV.	Normal	−	55	+
	Case 2	Male	34	Frameshift; c.2185dup	−	COVID 19	−	−	+	−	+	Subepicardial LGE in apical anterolateral and inferolateral walls	Severe LV dilatation	66	16	−
Lemus Barrios et al. (4)	Case	Male	49	Frameshift; c.6697_6698del p. Val2233Glnfs*2	−	−	+	+	−	+	+	LGE in the inferior and anterior segments in the LV	LV, RV dilatation	−	15	−
McColl et al. (17)	Case	Female	38	Frameshift; c.2848dupp. Ile950Asnfs*3	−	−	+	−	−	−	−	LGE in anterior wall in the LV	Normal	−	−	−

Literature	Case	Sex	Laboratory findngs				Recurrent elevation of troponin/frequency	Treatment	Follow-up
			cTn (ng/L)	NT-pro-BNP (ng/dL)	LDL-C (mg/dL)	CRP (mg/L)			
Kissopoulou et al. (15)	Elder twin brother (Proband)	Male	3,600	−	−	12	+/2 within 1 year	Beta-blockers, ACEI	No complications were observed during the 3-year follow-up; cardiac function was normal; cMRI showed extensive scarring.
	Younger twin brother	Male	6,000	−	−	−	−	Beta-blockers, ACEI	
Rezaei Bookani et al. (16)	Case 1	Female	693	−	−	Normal	−	Beta-blockers, ACEI, colchicine	Troponin levels were normal during follow-up visits; LVEF was stable during the 1-year follow-up; ICD was refused.
	Case 2	Male	Nomal	−	−	Normal	−	ICD, epicardial VT ablation, amiodarone, beta-blockers, ARB, MRA, SGLT2 inhibitors	LVEF remains low but stable during 1-year follow-up, with no further VT.
Lemus Barrios et al. (4)	Case	Male	Normal	318	86	−	−	ICD, metoprolol succinate, ARNI, spironolactone, dapagliflozin	Cardiovascular symptoms were disappeared, NT-proBNP was decreased to 115 ng/dL, LVEF improved to 43% during 7-month follow-up.
McColl et al. (17)	Case	Female	1,984	−	−	−	+/several times within 11 months	Colchicine, Prednisone, azathioprine, anakinra, prednisolone	Condition was control by immunosuppression, and an ICD was implanted due to high-risk factors.

VT, ventricular tachycardia; cMRI, cardiac magnetic resonance imaging; LVDd, left ventricular diastolic dimension; LVEF, left ventricular ejection fraction; cTN, cardiac troponin; NT-pro-BNP, NT pro B-type natriuretic peptide; LDL-C, low-density lipoprotein cholesterol; CRP, C-reactive protein; ACEI, angiotensin-converting enzyme inhibitor; ICD, implantable cardioverter defibrillator; ARB, angiotensin II receptor blocker; MRA, mineralocorticoid receptor antagonist; SGLT2, sodium-glucose cotransporter 2; ARNI, angiotensin receptor neprilysin inhibitor.

The desmoplakin encoded by the *DSP* gene is a core component of desmosomes, which firmly anchors the cytoskeletons of adjacent cardiomyocytes (4). It has been proposed that the link between *DSP* deficiency and inflammatory phenotypes involves disruption of desmosomal structural integrity (7). Specifically, truncating variants in the *DSP* gene may produce a truncated or structurally compromised desmoplakin protein. Such ultrastructural alterations in desmoplakin can lead to diverse pathological phenotypes, likely through compromised desmosomal integrity that weakens mechanical coupling between cardiomyocytes. This mechanical failure may activate innate immune pathways, ultimately driving myocardial remodeling and fibrosis (8, 9). From a pathophysiological perspective, alterations in desmosomal proteins triggered by various stimuli such as exercise, hypertension, or toxicity can promote the release of proinflammatory molecules, initiating and perpetuating an inflammatory response that results in myocardial injury (10). Concurrently, if the mutation itself possesses intrinsic properties that predispose to myocarditis, it may lower the threshold for triggering inflammatory events following secondary triggers. This suggests a potential mechanism underlying recurrent myocarditis caused by *DSP* gene variant. Previous studies have suggested that truncating *DSP* mutations are distributed relatively uniformly across the *DSP* coding sequence, whereas missense mutations may cluster in specific domains, such as the plakophilin/plakoglobin-binding region and the desmin-binding domain (11). In the context of missense mutations, the precise location of the variant may influence the resulting disease phenotype (12). In an 18-patient cohort with *DSP* gene mutations, cMRI identified LGE consistent with left ventricular myocardial fibrosis in all cases, including 2 individuals harboring *DSP* missense mutations (13). Additionally, a pair of twin brothers carrying a heterozygous missense mutation in the *DSP* gene both presented with chest pain and elevated troponin levels (14). Based on these observations, we reasonably hypothesize that, in addition to the well-established pathogenicity associated with truncating *DSP* mutations, missense variants in *DSP* may also be implicated in recurrent myocarditis. Future functional studies are warranted to determine whether the p.Ser766Thr variant specifically affects protein function.

## 4 Conclusion

This case report describes a 23-year-old female patient presenting with recurrent myocarditis, characterized by episodic chest pain and elevated serum troponin levels. cMRI confirmed the diagnosis by demonstrating LGE in the left ventricular anterior and anterolateral walls. WES identified a heterozygous missense variant in the *DSP* gene (c.2297G > C, p. Ser766Thr), which is classified as a VUS. The case underscores the consideration of genetic testing in young patients with unexplained recurrent troponin elevation, with the recognition that VUS findings require cautious interpretation and cannot establish causality. Whether specific missense variants, such as p.Ser766Thr, confer susceptibility to inflammatory myocardial injury remains to be determined through functional studies and validation in larger cohorts.

## Data Availability

The original contributions presented in the study are included in the article/Supplementary Material, further inquiries can be directed to the corresponding author.
